# Death of Dr. Graves

**Published:** 1853-06

**Authors:** 


### SELECTIONS

FROM

### FOREIGN AND HOME MEDICAL JOURNALS.

---

*Death of Dr. Graves.*—We record with profound regret the death of ROBERT JAMES GRAVES, of Dublin, which took place on the 20th of March, in the fifty-seventh year of his age. Dr. Graves was one of the brightest ornaments of the Dublin School, and his labors had made his name as familiar in America as it was in Ireland.

Dr. Graves was the youngest son of the Rev. Richard Graves, Dean of Ardagh. After having received a very careful preliminary education, he entered as a fellow-commoner in Trinity College, Dublin and at once evinced abilities of a high order. Among a large number of candidates at a July entrance, he took the first place, and from this time maintained a high status in his class, having been a "double first" man almost in his entire undergraduate course. With two exceptions he obtained the first prize in classics and science at every examination, and having put in every term examination, and obtained a *valdé in omnibus*, he thus entitled himself to. and received a gold medal, on taking his degree of Bachelor in Arts.

Having determined to devote himself to medicine, Mr. Graves commenced his professional studies in Dublin, and labored with equal zeal and industry in the acquirement of medical knowledge, as he had done previously in his university career.

Having graduated in medicine, he took the wise determination of visiting some of the chief schools of Europe, and of thus extending the education he had received at home by familiarizing himself with the modes of observation pursued elsewhere. After a short stay in London, where he studied under Sir William Blizard and Dr. Robinson, he spent three years in visiting the chief Continental Schools, among which may be mentioned those of Berlin, Gottingen, Hamburgh, Copenhagen, etc. Among the distinguished teachers with whom Dr. Graves was now brought into contact, were Hufeland and Behrend, under whom he acquired that taste for the clinical study of disease, and the cultivation of pathology, which so strongly marked his subsequent career, and so characterize his writings.

In the year 1821, Dr. Graves settled in Dublin, and having succeeded, in conjunction with some other surgeons and physicians, in establishing a private school of medicine, and having also been elected one of the physicians of the Meath, entered with great energy and zeal on the arduous career of a medical teacher. The school of which he was one of the founders, known as the Park-street School, (now the site of St. Mark's Ophthalmic Hospital,) rapidly acquired a very high character. Here he first taught medical jurisprudence, subsequently pathological anatomy, but afterwards became associated with Doctor, now Sir Henry Marsh, in the Chair of Practice of Physic. The Meath Hospital, however, was the great theatre of his most important labors.

Here he found apt and zealous pupils, and many of the best and most accomplished practitioners now in Ireland, England, the Colonies and the public service, were then numbered among Dr. Graves' class at the Meath Hospital, and many have lived to acknowledge with pleasure and pride the obligations they owed to his teachings, and the stimulus which his example lent to their exertions. Two among this number must be specially named: Dr. Richard Townsend and Dr. William Stokes, now Regius Professor of Physic in the University of Dublin.

Thus energetically and ardently working and teaching, the example of Dr. Graves at this period exercised the best influence on the medical youth of Dublin. His labors, however, were not confined to those of teaching. From an early period in his medical career he evinced the highest talents for original observation, and the results of his inquiries began to appear in print, and to attract attention, from the masterly style of his delineations of disease, his graphic manner, and the clearness, judgment and decision with which his views were enunciated.

Dr. William Stokes having graduated in Edinburgh, and having subsequently been appointed Dr. Graves' colleague at the Meath Hospital, these two names are henceforth to be met with together as teachers and fellow laborers in the field of original research. Under their joint editorship appeared the valuable series of Meath Hospital Reports, which have connected the name of this institution with the progress of Irish medicine during the last thirty years.

In the year 1827, Dr. Graves was elected Professor of the Institutes of Medicine to the King and Queen's College of Physicians in Ireland—a chair which he continued to fill for many years with great distinction.

Independently of the publication of his various detached papers and monographs, Dr. Graves lent valuable assistance to the establishment of a periodical medical literature in Ireland. In the year 1832, the fifth and last volume of the "Dublin Hospital Reports" was committed to his editorship by Dr. Cheyne. Two years subsequently, the *Dublin Journal of Medical and Chemical Science*, the predecessor of the *Dublin Quarterly Journal of Medicine*, was projected and established by Sir Robert Kane, then a student of Medicine and a pupil at the Meath Hospital. After the appearance of a few Numbers, Drs. Graves and Stokes became associated in the editorship of the periodical, and Dr. K ne having been soon forced to resign his connection with it by his increasing devotion to chemical inquiry, it continued in the same hands till 1842.

In the original and review department of this journal, Dr. Graves was a large and constant contributor. In the year 1843, appeared the first edition of his "Clinical Lectures on the Practice of Medicine." Of this work, which passed through a second edition, with much careful revision, and the addition of much valuable matter under the hands of Dr. Neligan, in the year 1848, it is quite unnecessary for us to speak. It is well known to every clinical school in Europe.

As a lecturer, Dr. Graves was distinguished by a force and clearness of language, and an earnestness of manner which irresistibly commanded attention, while his fine person and noble features won the admiration of his hearers.

His style as a writer was at once simple yet nervous, and full of graphic power; and his delineations of disease are among the most successful of modern medical compositions. He was a strenuous advocate of the doctrine of contagion, and vigorously opposed the views advanced with regard to the non contagious nature of cholera during its last outbreak. He was a firm supporter of the dignity and honor of his profession, and on the only occasion in which he descended into the arena of medical politics, he fought boldly and fearlessly, though unsuccessfully, for the rights of his brother proctitioners. During many years, Dr. Graves enjoyed a large and lucrative practice, and was much *recherché* as a consultant. For some time past his health has been only indifferent, and of late he suffered from attacks of atonic gout. His last illness was attended with considerable suffering, the lungs being much congested. He had also violent paroxysmal attacks of cough, which the slightest exertion was sufficient to induce. Symptoms of a purpuric condition of the blood, accompanied by anasarca, were subsequently manifested. —*Lancet and Med. Times and Gazette.—Med. News.*

